# Prevalence and Genetic Analysis of Thalassemia and Hemoglobinopathy in Different Ethnic Groups and Regions in Hainan Island, Southeast China

**DOI:** 10.3389/fgene.2022.874624

**Published:** 2022-06-13

**Authors:** Min Wang, Xiaozhuang Zhang, Yubin Zhang, Meifang Xiao

**Affiliations:** ^1^ Hainan Women and Children’ Medical Center, Haikou, China; ^2^ NHC Key Laboratory of Tropical Disease Control, Hainan Medical University, Haikou, China

**Keywords:** prevalence, childbearing age, li nationality, Hainan, thalassemia

## Abstract

**Background:** There are limited studies on the molecular profile of thalassemia in Hainan, the free trade island in China. Our aim was to reveal the prevalence and molecular mutation spectrum of thalassemia in different ethnic groups and regions of Hainan through a large sample study for the first time.

**Methods:** A total of 231,596 individuals from 19 cities and counties in Hainan were screened by hematological parameter analysis, and further genetic analysis was performed on individuals with MCV less than 82 fL.

**Results:** Totally, 31,780 (13.72%) subjects were diagnosed as thalassemia carriers. The overall prevalence of α-thalassemia, β-thalassemia, and α+β-thalassemia were 11.04%, 1.48%, and 1.20%, respectively. We further analyzed the molecular profiles of thalassemia in various ethnic groups and mainly compared the difference between Han and Li. The results showed that the frequency of thalassemia in the Li population (47.03%) was much higher than that in Han (9.37%). Except for β-thalassemia (1.31% of Li vs. 1.47% of Han), the frequencies of α-thalassemia (39.59% of Li vs. 7.35% of Han) and α+β-thalassemia (6.13% of Li vs. 0.56% of Han) in the Li were obviously higher than those in Han. The high-frequent genotypes of α-thalassemia in Han were αα/--^SEA^ (25.55%), -α^3.7^/αα (22.17%), -α^4.2^/αα (21.59%), α^WS^α/αα (8.93%), and -α^3.7^/-α^4.2^ (4.17%) and those of Li were -α^4.2^/αα (17.24%), -α^3.7^/αα (17.16%), -α^3.7^/-α^4.2^ (15.09%), α^WS^α/αα (9.69%), and α^WS^α/-α^3.7^ (8.06%), respectively. The αα/--^SEA^ was the highest genotype of α-thalassemia in Han but only accounted for 1.87% in Li. For β-thalassemia, the top three high-frequent genotypes in both Han and Li were β^CD41/42(-TTCT)^/β^N^, β^-28(A>G)^/β^N^, and β^IVS-Ⅱ-654(C>T)^/β^N^, but the frequency of β^CD41/42(-TTCT)^/β^N^ in Li (90.96%) was much higher than that in Han (56.32%) and the data reported in other provinces of China. Additionally, the prevalence of thalassemia ranged from 8.16% to 34.35% in Hainan, Wuzhishan, Baoting, Qiongzhong, and Baisha have a higher prevalence than other areas.

**Conclusion:** Our study revealed the characteristics of ethnic and regional differences in the prevalence of thalassemia in the childbearing age population of Hainan for the first time, indicating that the prevalence of thalassemia among Li nationality is the highest in China. Those findings will be useful for genetic counseling and the prevention of thalassemia.

## Introduction

Thalassemia is one of the most widely distributed autosomal monogenic diseases in the world. It was first found in Italy, Malta, Greece, and other regions along the Mediterranean coast. The southern part of the Yangtze River in China is also the high-risk area for this disease, among which the prevalence of Guangxi (19.10%), Hainan (12.95%), and Guangdong (11.90%) is more than 10%. (He et al., 2017; He et al., 2018; Peng et al., 2021; Wang et al., 2022). At the molecular level, according to the type of globin gene defect involved, thalassemia can be divided into several subtypes; the most common subtypes are α-thalassemia and β-thalassemia. The coding gene of α-globin is located on chromosome 16 with two linked α genes on each homologous chromatid. Depending on whether one or both of the linked α genes are deleted or less active due to mutations, α-gene defects can be classified into α^+^ and α^0^. (Piel and Weatherall, 2014). The coding gene of β-globin is located on chromosome 11, and its defects include point mutations and deletions, and point mutations are the most common. Currently, over 200 types of β-gene defects have been identified, ranging from mild mutations that result in a relative reduction in β-globin peptide chain synthesis (β^+^) to severe mutations that completely inhibit β-globin peptide chain synthesis (β^0^). (Needs et al., 2022). Patients with HbH disease (α^0^/α^+^) can develop mild to moderate anemia with age and may require a blood transfusion, while patients with the α-thalassemia major genotype (α^0^/α^0^) usually die during the fetal stage or shortly after birth. Patients with β-thalassemia major or some β-thalassemia intermedia genotypes (β^+^/β^+^ or β^0^/β^+^ or β^0^/β^0^) rely on transfusion to sustain their life. (Taher et al., 2018). Therefore, the birth of children with severe thalassemia will bring heavy mental and economic burdens to their families and society.

Hainan Island, a region located in the southernmost part of China, covers an area of 35,400 square kilometers and contains 19 cities and counties, with an estimated population of about 10 million. Hainan Island is also a multi-ethnic settlement, with a population mainly composed of Han and Li people, and a minority of the Miao, Zhuang, and Hui people, among which the Li people are the earliest residents of Hainan Island mainly living in Lingshui, Baoting, Baisha, Qiongzhong, Ledong, and Changjiang. People of childbearing age refer to individuals who have the ability to bear children, usually between the age of 15 and 50 for women and from 16 to 65 for men. In order to prevent and control severe thalassemia, the Hainan provincial government has implemented the “Hainan Pregnancy Thalassemia Screening Program” since 2019 to provide a free genetic diagnosis of thalassemia for pregnant women and their partners. Our previous study showed that the overall prevalence of thalassemia in Hainan was as high as 12.95%. (Wang et al., 2022). However, the prevalence and molecular mutation spectrum of thalassemia in different ethnic groups and regions have not been reported. In this study, we used the data obtained from this project to characterize the prevalence and molecular spectrum of thalassemia in different ethnic groups and regions in Hainan, indicating that the prevalence of thalassemia in Hainan has obvious ethnic and regional differences. Those findings will be useful for genetic counseling and the prevention of thalassemia.

## Materials and Methods

### Participants

The subjects of this study were couples who underwent prenatal health check-ups in medical institutions in 19 cities and counties in Hainan between January 2020 and December 2021. Both couples were included in the study after signing informed consent. All participants’ sex, age, nationality, and domicile were available to ensure a comprehensive understanding of the research subjects. All participants voluntarily joined this study with informed consent, and all studies were approved by the Ethics Committee for Clinical Investigation of Hainan Women and Children’s Medical Center.

### Hematological Parameter Analysis

All subjects were recruited from the medical institutions within 19 cities and counties in Hainan. Peripheral venous blood samples of 2 ml volume were taken from all subjects and stored in EDTA anti-coagulated tubes. The hematology phenotypic indicators were determined and analyzed by the medical institutions where the subjects were located using the hemocyte analyzer. Subjects with MCV values less than 82 fL were considered possible thalassemia carriers.

### Gene Diagnosis of Thalassemia

For the diagnosis, 2 ml of peripheral blood of the participants was collected with an EDTA anticoagulant tube and transported to Hainan Women and Children’s Medical Center and Sanya Women and Children’s Hospital by cold-chain immediately for gene diagnosis of thalassemia. The temperature of the cold chain was 4–8°C. The specimen transport was generally completed within 2–5 days and no more than 7 days from blood collection to the specimen received by the laboratory. The gap polymerase chain reaction (Gap-PCR) was used to identify three common Chinese α-globin gene deletion mutations (–α^3.7^, –α^4.2^, – –^SEA^) and rare deletion mutations (#20193401915, Yaneng Biosciences, Shenzhen, China). Reverse dot-blot hybridization was used to identify three common nondeletional mutations of α-thalassemia (Hb CS, Hb QS, and Hb WS) (#20173401107, Yaneng Biosciences, Shenzhen, China) and 17 common β-globin gene mutations in China (#20163400463, Yaneng Biosciences, Shenzhen, China). The DNA sequence was used to detect rare and unknown thalassemia gene mutations.

### Statistics

Excel 2016 and GraphPad Prism 8 (GraphPad Software, La Jolla, CA) were used for statistical analyses. The comparison of composition ratios between two or more groups using Fisher’s exact test or chi-squared test and multiple comparisons of composition ratios among different groups were determined using the Bonferroni method. Values of *p* < 0.05 were considered statistically significant.

## Results

### Molecular Epidemiological Characteristics of Thalassemia Genotypes in Childbearing Age Population of Hainan

In this study, a total of 231,596 individuals from 19 cities and counties in Hainan were screened by hematological parameter analysis, and genetic analysis was performed on individuals with MCV less than 82 fL. A total of 31,780 (13.72%) subjects were diagnosed as carriers or patients of thalassemia. The overall prevalence of α-thalassemia, β-thalassemia, and α+β-thalassemia were 11.04%, 1.48%, and 1.20%, respectively. Among α-thalassemia carriers, a total of 33 genotypes were detected in 25,578 α-thalassemia carriers. The high-frequent genotypes of α-thalassemia were -α^3.7^/αα, -α^4.2^/αα, αα/--^SEA^, α^WS^α/αα, and -α^3.7^/-α^4.2^, accounting for 20.16%, 19.62%, 16.13%, 9.31%, and 8.55% of all α-thalassemia genotypes in turn (Table 1). A total of 16 genotypes were found in 3,417 β-thalassemia carriers, and β^CD41/42 (-TTCT)^/β^N^ was the most frequent genotype, accounting for 59.91% of all β-thalassemia genotypes. Other high-prevalent genotypes of β-thalassemia were β^-28(A>G)^/β^N^ (16.21%), β^IVS-Ⅱ-654(C>T)^/β^N^ (8.08%), β^CD71/72(+A)^/β^N^ (5.62%), β^CD17(A>T)^/β^N^ (5.44%), and β^CD26 (GAG>AAG)^/β^N^ (2.66%); the aforementioned six genotypes accounted for 97.92% of all β-thalassemia genotypes in Hainan (Table 2). In total, 2,785 subjects were diagnosed with α+β-thalassemia, and among them, the high-frequent genotypes were -α^3.7^/αα combined with β^CD41/42 (-TTCT)^/β^N^, -α^4.2^/αα combined with β^CD41/42 (-TTCT)^/β^N^, and α^WS^α/αα combined with β^CD41/42 (-TTCT)^/β^N^, accounting for 20.00%, 19.17%, and 12.96% of all α+β-thalassemia genotypes, respectively (Supplementary Table S1). In addition, this study also identified some uncommon thalassemia genotypes in the Chinese population. Rare α-thalassemia genotypes were composed of Fusion/-α^4.2^ (16 cases), Fusion/-α^3.7^ (14 cases), HKαα/αα (8 cases), HKαα/--^SEA^ (3 cases), αααanti^4.2^∕-α^3.7^ (3 cases), --^THAI^ (1 case), -α^3.7^/α^IVS-II-55,IVS-II-119^α (1 case), -α^4.2^/α^IVS-II-55,IVS-II-119^α (1 case), and α^WS^α/HKαα (1 case). Two rare β-thalassemia genotypes of β^IVS-I-1 (G>T)^/β^IVS-Ⅱ-81 (C>T)^ and β^5’UTR;+40–43 (A>C)^/β^IVS-Ⅱ-81 (C>T)^ were also identified. Furthermore, two cases with β^-50 (G>A)^/β^N^ were found in all α+β-thalassemia genotypes (Supplementary Table S1).

**TABLE 1:** Molecular mutation spectrum of α-thalassemia in the population of childbearing age in the Hainan Island.

α-thalassemia genotype	Type	All number of cases (n)	Frequency of all (%)	Cases of Han (n)	Frequency of Han (%)	Cases of Li (n)	Frequency of Li (%)
**αα/--** ^**SEA**^	**α** ^**0**^ **/α**	**4,125**	**16.13**	**3,766**	**25.55**	**195**	**1.87**
**αα/--** ^**THAI**^	**α** ^**0**^ **/α**	**1**	**0.00**	**1**	**0.01**	**0**	**0.00**
**-α** ^**3.7**^ **/--** ^**SEA**^	**α** ^**+**^ **/α** ^**0**^	**248**	**0.97**	**175**	**1.19**	**66**	**0.63**
**-α** ^**3.7**^ **/-α** ^**3.7**^	**α** ^**+**^ **/α** ^**+**^	**1,081**	**4.23**	**297**	**2.02**	**775**	**7.45**
**-α** ^**3.7**^ **/-α** ^**4.2**^	**α** ^**+**^ **/α** ^**+**^	**2,188**	**8.55**	**615**	**4.17**	**1,570**	**15.09**
**-α** ^**3.7**^ **/αα**	**α** ^**+**^ **/α**	**5,157**	**20.16**	**3,268**	**22.17**	**1,785**	**17.16**
**-α** ^**4.2**^ **/--** ^**SEA**^	**α** ^**+**^ **/α** ^**0**^	**204**	**0.80**	**138**	**0.94**	**62**	**0.60**
**-α** ^**4.2**^ **/-α** ^**4.2**^	**α** ^**+**^ **/α** ^**+**^	**1,059**	**4.14**	**315**	**2.14**	**740**	**7.11**
**-α** ^**4.2**^ **/αα**	**α** ^**+**^ **/α**	**5,019**	**19.62**	**3,182**	**21.59**	**1,793**	**17.24**
**α** ^**CS**^ **α/--** ^**SEA**^	**α** ^**+**^ **/α** ^**0**^	**16**	**0.06**	**14**	**0.09**	**1**	**0.01**
**α** ^**CS**^ **α/-α** ^**3.7**^	**α** ^**+**^ **/α** ^**+**^	**42**	**0.16**	**33**	**0.22**	**8**	**0.08**
**α** ^**CS**^ **α/-α** ^**4.2**^	**α** ^**+**^ **/α** ^**+**^	**40**	**0.16**	**25**	**0.17**	**15**	**0.14**
**α** ^**CS**^ **α/αα**	**α** ^**+**^ **/α**	**141**	**0.55**	**121**	**0.82**	**4**	**0.04**
**α** ^**QS**^ **α/--** ^**SEA**^	**α** ^**+**^ **/α** ^**0**^	**12**	**0.05**	**10**	**0.07**	**2**	**0.02**
**α** ^**QS**^ **α/-α** ^**3.7**^	**α** ^**+**^ **/α** ^**+**^	**160**	**0.63**	**45**	**0.31**	**115**	**1.11**
**α** ^**QS**^ **α/-α** ^**4.2**^	**α** ^**+**^ **/α** ^**+**^	**137**	**0.54**	**37**	**0.25**	**100**	**0.96**
**α** ^**QS**^ **α/αα**	**α** ^**+**^ **/α**	**808**	**3.16**	**583**	**3.96**	**218**	**2.10**
**α** ^**WS**^ **α/--** ^**SEA**^	**α** ^**+**^ **/α** ^**0**^	**143**	**0.56**	**96**	**0.65**	**39**	**0.37**
**α** ^**WS**^ **α/-α** ^**3.7**^	**α** ^**+**^ **/α** ^**+**^	**1,134**	**4.43**	**294**	**1.99**	**839**	**8.06**
**α** ^**WS**^ **α/-α** ^**4.2**^	**α** ^**+**^ **/α** ^**+**^	**1,025**	**4.01**	**280**	**1.90**	**742**	**7.13**
**α** ^**WS**^ **α/αα**	**α** ^**+**^ **/α**	**2,382**	**9.31**	**1,316**	**8.93**	**1,008**	**9.69**
**α** ^**CS**^ **α/α** ^**WS**^ **α**	**α** ^**+**^ **/α** ^**+**^	**6**	**0.02**	**4**	**0.03**	**1**	**0.01**
**α** ^**QS**^ **α/α** ^**WS**^ **α**	**α** ^**+**^ **/α** ^**+**^	**88**	**0.34**	**25**	**0.17**	**63**	**0.61**
**α** ^**QS**^ **α/α** ^**QS**^ **α**	**α** ^**+**^ **/α** ^**+**^	**9**	**0.04**	**4**	**0.03**	**5**	**0.05**
**α** ^**WS**^ **α/α** ^**WS**^ **α**	**α** ^**+**^ **/α** ^**+**^	**306**	**1.20**	**65**	**0.44**	**240**	**2.31**
**Fusion/-α** ^**3.7**^	**α** ^**+**^ **/α** ^**+**^	**14**	**0.05**	**5**	**0.03**	**9**	**0.09**
**Fusion/-α** ^**4.2**^	**α** ^**+**^ **/α** ^**+**^	**16**	**0.06**	**8**	**0.05**	**8**	**0.08**
**HKαα/αα**	**α** ^**+**^ **/α**	**8**	**0.03**	**8**	**0.05**	**0**	**0.00**
**HKαα/--** ^**SEA**^	**α** ^**+**^ **/α** ^**0**^	**3**	**0.01**	**3**	**0.02**	**0**	**0.00**
**αααanti** ^**4.2**^ **/-α** ^**3.7**^	**α** ^**+**^ **/α** ^**+**^	**3**	**0.01**	**3**	**0.02**	**0**	**0.00**
**α** ^**WS**^ **α/HKαα**	**α** ^**+**^ **/α** ^**+**^	**1**	**0.00**	**1**	**0.01**	**0**	**0.00**
**-α** ^**3.7**^ **/α** **^a^** ^**, #**^ **α**	**α** ^**+**^ **/α**	**1**	**0.00**	**1**	**0.01**	**0**	**0.00**
**-α** ^**4.2**^ **/α** **^a^** ^**, #**^ **α**	**α** ^**+**^ **/α**	**1**	**0.00**	**1**	**0.01**	**0**	**0.00**
**Total**		**25,578**	**100.00**	**14,739**	**100.00**	**10,403**	**100.00**

a IVS-II-55 (T > G); #: IVS-II-119 (-G,+CTCGGCCC).

n, number.

**TABLE 2:** Molecular mutation spectrum of β-thalassemia in the population of childbearing age in the Hainan Island.

β-thalassemia genotype	Type	All number of cases (n)	Frequency of all (%)	Cases of Han (n)	Frequency of Han (%)	Cases of Li (n)	Frequency of Li (%)
**β** ^CD14/15 (+G)^ **/β** ^N^	**β** ^0^ **/β**	**4**	**0.12**	**4**	**0.14**	**0**	**0.00**
**β** ^CD17 (A > T)^ **/β** ^N^	**β** ^0^ **/β**	**186**	**5.44**	**159**	**5.41**	**2**	**0.58**
**β** ^CD27/28 (+C)^ **/β** ^N^	**β** ^0^ **/β**	**11**	**0.32**	**9**	**0.31**	**1**	**0.29**
**β** ^-28 (A > G)^ **/β** ^N^	**β** ^+^ **/β**	**554**	**16.21**	**532**	**18.11**	**10**	**2.92**
**β** ^-29 (A > G)^ **/β** ^N^	**β** ^+^ **/β**	**14**	**0.41**	**12**	**0.41**	**0**	**0.00**
**β** ^CD41/42 (-TTCT)^ **/β** ^N^	**β** ^0^ **/β**	**2,047**	**59.91**	**1,654**	**56.32**	**312**	**90.96**
**β** ^CD43 (G > T)^ **/β** ^N^	**β** ^0^ **/β**	**7**	**0.20**	**7**	**0.24**	**0**	**0.00**
**β** ^IVS-Ⅱ-654 (C > T)^ **/β** ^N^	**β** ^+^ **/β**	**276**	**8.08**	**267**	**9.09**	**8**	**2.33**
**β** ^CD71/72 (+A)^ **/β** ^N^	**β** ^0^ **/β**	**192**	**5.62**	**183**	**6.23**	**4**	**1.17**
**β** ^5′UTR; +40–43 (A > C)^ **/β** ^N^	**β** ^+^ **/β**	**1**	**0.03**	**1**	**0.03**	**0**	**0.00**
**β** ^Init CD (ATG > AGG)^ **/β** ^N^	**β** ^0^ **/β**	**16**	**0.47**	**15**	**0.51**	**1**	**0.29**
**β** ^IVS-I-1 (G > T)^ **/β** ^N^	**β** ^0^ **/β**	**14**	**0.41**	**11**	**0.37**	**0**	**0.00**
**β** ^IVS-I-5 (G > C)^ **/β** ^N^	**β** ^+^ **/β**	**2**	**0.06**	**2**	**0.07**	**0**	**0.00**
**β** ^CD26 (GAG > AAG)^ **/β** ^N^	**β** ^+^ **/β**	**91**	**2.66**	**81**	**2.76**	**5**	**1.46**
**β** ^CD27/28 (+C)^ **/β** ^&^	**β** ^0^ **/β**	**1**	**0.03**	**0**	**0.00**	**0**	**0.00**
**β** ^IVS-I-1 (G > T)^ **/β**&	**β** ^0^ **/β**	**1**	**0.03**	**0**	**0.00**	**0**	**0.00**
**Total**		**3,417**	**100.00**	**2,937**	**100.00**	**343**	**100.00**

&: IVS-Ⅱ-81 (C > T).

n, number.

We further calculated the frequency of specific mutation in all α (or β) mutant chromosomes (allele frequency) in Hainan. Twelve α-thalassemia gene mutations and sixteen β-thalassemia gene mutations were identified. Among the 12 α-gene mutations, the six top frequent types were -α^3.7^, -α^4.2^, α^WS^α, --^SEA^, and α^QS^α, with the allele frequencies of 33.35%, 32.23%, 16.67%, 13.35%, and 3.54% of all α mutant chromosomes in turn. Other mutations with allele frequencies less than 1% included α^CS^α, fusion, HKαα, αααanti^4.2^, --^THAI^, α^IVS-II-55 (T>G)^α, and α^IVS−II−119 (-G,+CTCGGCCC)^α (Table 3). Among the 16 β-gene mutations, β^CD41/42(-TTCT)^ had the highest allele frequency of 73.08%, followed by β^-28 (A>G)^ (10.84%), β^IVS-Ⅱ-654 (C>T)^ (5.25%), β^CD71/72 (+A)^ (3.96%), β^CD17 (A>T)^ (3.62%), and β^CD26 (GAG>AAG)^ (1.71%). Other mutations with allele frequencies less than 1% included β^CD14/15 (+G)^, β^CD27/28 (+C)^, β^-29 (A>G)^, β^CD43 (G>T)^, β^5’UTR;+40–43 (A>C)^, β^Init CD (ATG>AGG)^, β^IVS-I-1 (G>T)^, β^IVS-I-5 (G>C)^, β^-50 (G>A)^, and β^IVS-Ⅱ-81 (C>T)^ (Table 4).

**TABLE 3:** Allele frequency of α-thalassemia mutations in the population of childbearing age in the Hainan Island.

α-thalassemia gene	Type	All alleles (n)	Frequency of all (%)	Allele of Han (n)	Frequency of Han (%)	Allele of Li (n)	Frequency of Li (%)
**-α** ^**3.7**^	**α** ^**+**^	**12,410**	**33.35**	**5,464**	**29.47**	**5,918**	**36.67**
**-α** ^**4.2**^	**α** ^**+**^	**11,993**	**32.23**	**5,333**	**28.76**	**5,760**	**35.69**
**--** ^**SEA**^	**α** ^**0**^	**4,968**	**13.35**	**4,360**	**23.52**	**404**	**2.50**
**α** ^**CS**^ **α**	**α** ^**+**^	**262**	**0.70**	**210**	**1.13**	**29**	**0.18**
**α** ^**QS**^ **α**	**α** ^**+**^	**1,319**	**3.54**	**737**	**3.97**	**570**	**3.53**
**α** ^**WS**^ **α**	**α** ^**+**^	**6,201**	**16.67**	**2,401**	**12.95**	**3,441**	**21.32**
**--** ^**THAI**^	**α** ^**0**^	**1**	**0.00**	**0**	**0.00**	**0**	**0.00**
**α** ^**IVS-II−55 (T**>**G)**^ **α**	**α**	**2**	**0.01**	**2**	**0.01**	**0**	**0.00**
**α** ^**IVS-II−119 (#)**^ **α**	**α**	**2**	**0.01**	**2**	**0.01**	**0**	**0.00**
**Fusion**	**α** ^**+**^	**32**	**0.09**	**14**	**0.08**	**18**	**0.11**
**HKαα**	**α** ^**+**^	**14**	**0.04**	**14**	**0.08**	**0**	**0.00**
**αααanti** ^**4.2**^	**α** ^**+**^	**4**	**0.01**	**4**	**0.02**	**0**	**0.00**
**Total**		**37,208**	**100.00**	**18,541**	**100.00**	**16,140**	**100.00**

#: -G,+CTCGGCCC.

n, number.

**TABLE 4:** Allele frequency of β-thalassemia mutations in the population of childbearing age in the Hainan Island.

β-thalassemia gene	Type	All alleles (n)	Frequency of all (%)	Allele of Han (n)	Frequency of Han (%)	Allele of Li (n)	Frequency of Li (%)
**CD14/15 (+G)**	**β** ^**+**^	**4**	**0.06**	**4**	**0.10**	**0**	**0.00**
**CD17 (A** > **T)**	**β** ^**0**^	**225**	**3.62**	**189**	**4.66**	**2**	**0.10**
**CD27/28 (+C)**	**β** ^**0**^	**14**	**0.23**	**11**	**0.27**	**1**	**0.05**
**-28 (A** > **G)**	**β** ^**+**^	**673**	**10.84**	**639**	**15.75**	**21**	**1.07**
**-29 (A** > **G)**	**β** ^**+**^	**18**	**0.29**	**16**	**0.39**	**0**	**0.00**
**CD41/42 (-TTCT)**	**β** ^**0**^	**4,537**	**73.08**	**2,523**	**62.17**	**1,891**	**96.73**
**CD43 (G** > **T)**	**β** ^**0**^	**13**	**0.21**	**13**	**0.32**	**0**	**0.00**
**IVS-Ⅱ-654 (C** > **T)**	**β** ^**0**^	**326**	**5.25**	**309**	**7.61**	**15**	**0.77**
**CD71/72 (+A)**	**β** ^**0**^	**246**	**3.96**	**224**	**5.52**	**15**	**0.77**
**5′UTR;+40–43 (A** > **C)**	**β** ^**+**^	**4**	**0.06**	**4**	**0.10**	**0**	**0.00**
**Init CD (ATG** > **AGG)**	**β** ^**0**^	**20**	**0.32**	**19**	**0.47**	**1**	**0.05**
**IVS-I-1 (G** > **T)**	**β** ^**0**^	**16**	**0.26**	**12**	**0.30**	**0**	**0.00**
**IVS-I-5 (G** > **C)**	**β** ^**+**^	**2**	**0.03**	**2**	**0.05**	**0**	**0.00**
**CD26 (GAG** > **AAG)**	**β** ^**+**^	**106**	**1.71**	**93**	**2.29**	**7**	**0.36**
**-50 (G** > **A)**	**β** ^**+**^	**2**	**0.03**	**0**	**0.00**	**2**	**0.10**
**IVS-Ⅱ-81 (C** > **T)**	**β**	**2**	**0.03**	**0**	**0.00**	**0**	**0.00**
**Total**		**6,208**	**100.00**	**4,058**	**100.00**	**1,955**	**100.00**

n, number.

### Molecular Epidemiological Characteristics of Thalassemia Genotypes in Han and Li Nationalities of Childbearing Age Population in Hainan

A total of 31,780 thalassemia gene carriers were identified in this study, among which 59.13% were Han people, 38.89% were Li people, and 1.98% were from other ethnic groups. Then, we analyzed the prevalence and the molecular spectrum of thalassemia in different ethnic groups; the prevalence of thalassemia in various ethnic groups is shown in Table 5, and the molecular profiles of thalassemia in Miao, Zhuang, and Hui are shown in Supplementary Tables S4–S8. Due to the small number of subjects included in the total screening of Miao, Zhuang, and Hui, the prevalence of thalassemia in those groups may be inaccurate. Therefore, we mainly compared the differences in the molecular mutation spectrum of thalassemia genotypes between Han and Li populations. The results showed that a total of 18,793 (9.37%) thalassemia carriers were detected among 200,467 Han population of childbearing age. The prevalence of α-thalassemia, β-thalassemia, and α+β-thalassemia were 7.35%, 1.47%, and 0.56%, respectively. The high-frequent genotypes of α-thalassemia were αα/--^SEA^ (25.55%), -α^3.7^/αα (22.17%), -α^4.2^/αα (21.59%), α^WS^α/αα (8.93%), and -α^3.7^/-α^4.2^ (4.17%), and those of β-thalassemia were β^CD41/42(-TTCT)^/β^N^ (56.32%), β^-28(A>G)^/β^N^ (18.11%), β^IVS-Ⅱ-654(C>T)4^/β^N^ (9.09%), β^CD71/72(+A)^/β^N^ (6.23%), and β^CD17(A>T)^/β^N^ (5.41%) (Tables 1, 2). A total of 12,358 (47.03%) thalassemia carriers were found among 26,277 Li population of childbearing age. The prevalences of α-thalassemia, β-thalassemia, and α+β-thalassemia were 39.59%, 1.31%, and 6.13%, respectively. The high-frequent genotypes of α-thalassemia were -α^4.2^/αα (17.24%), -α^3.7^/αα (17.16%), -α^3.7^/-α^4.2^ (15.09%), α^WS^α/αα (9.69%), αWSα/-α3.7 (8.06%), -α^3.7^/-α^3.7^ (7.45%), αWSα/-α4.2 (7.13%), and -α^4.2^/-α^4.2^ (7.11%), and those of β-thalassemia were β^CD41/42(-TTCT)^/β^N^ (90.96%), β^−28(A>G)^/β^N^ (2.92%), β^IVS-Ⅱ-654(C>T)4^/β^N^ (2.33%), β^CD26 (GAG>AAG)^/β^N^ (1.46%), and β^CD71/72(+A)^/β^N^ (1.17%) (Tables 1, 2). In both Han and Li nationalities, the high-frequent genotypes of α+β thalassemia were -α^3.7^/αα combined with β^CD41/42 (-TTCT)^/β^N^ (20.86% of Han vs. 19.35% of Li), -α^4.2^/αα combined with β^CD41/42 (-TTCT)^/β^N^ (18.98% of Han vs. 19.67% of Li), and α^WS^α/αα combined with β^CD41/42 (-TTCT)^/β^N^ (12.26% of Han vs. 13.46% of Li) (Supplementary Tables S2, S3).

**TABLE 5:** Prevalence of thalassemia in various ethnic groups in the population of childbearing age in the Hainan Island.

Nationality	Total number of screening (n)	Prevalence of thalassemia (%)	Prevalence of α-thalassemia (%)	Prevalence of β-thalassemia (%)	Prevalence of α+β-thalassemia (%)
**All**	**231,596**	**13.72**	**11.04**	**1.48**	**1.20**
**Han**	**200,467**	**9.37**	**7.35**	**1.47**	**0.56**
**Li**	**26,277**	**47.03**	**39.59**	**1.31**	**6.13**
**Miao**	**2,055**	**16.06**	**11.29**	**3.11**	**1.65**
**Zhuang**	**912**	**14.69**	**9.98**	**3.95**	**0.77**
**Hui**	**440**	**10.91**	**8.86**	**1.82**	**0.23**

n, number.

We further calculated the frequency of specific mutation in all α (or β) mutant chromosomes in both Han and Li nationalities. The results showed that the ranking of the high-frequent gene mutations of α-thalassemia was significantly different between the two nationalities. The six top frequent types of Han were -α^3.7^ (29.47%), -α^4.2^ (28.76%), --^SEA^ (23.52%), α^WS^α (12.95%), α^QS^α (3.97%), and α^CS^α (1.13%), and those of Li were -α^3.7^ (36.67%), -α^4.2^ (35.69%), α^WS^α (21.32%), α^QS^α (3.53%), --^SEA^ (2.50%), and α^CS^α (0.18%) (Table 3). Of β-gene mutant chromosomes, β^CD41/42(-TTCT)^ had the highest allele frequency in both Han (62.17%) and Li (96.73%) nationalities. Other high-frequent mutations were β^−28(A>G)^ (15.75% of Han vs. 1.07% of Li), β^IVS-Ⅱ-654(C>T)^ (7.61% of Han vs. 0.77% of Li), β^CD71/72 (+A)^ (5.52% of Han vs. 0.77% of Li), β^CD17(A>T)^ (4.66% of Han vs. 0.10% of Li), and β^CD26 (GAG>AAG)^ (2.29% of Han vs. 0.36% of Li) (Table 4).

### Prevalence of Thalassemia in Different Regions of the Hainan Island

**FIGURE 1:**
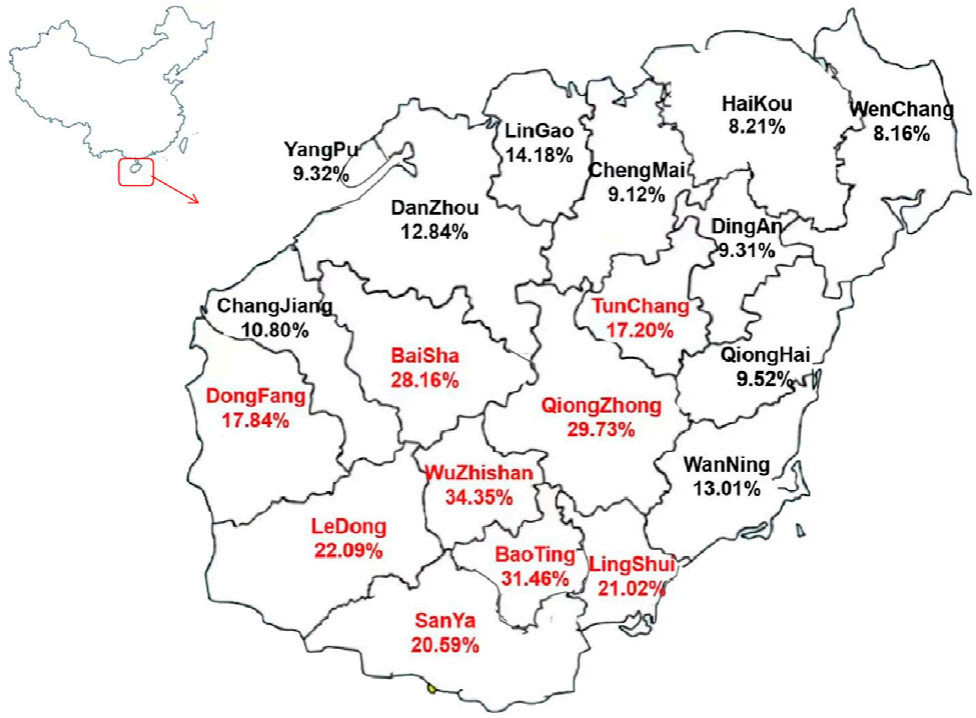
Prevalence of thalassemia in different regions of the Hainan Island.

The prevalence of thalassemia in 19 cities and counties of the Hainan Island was analyzed. The results showed that the prevalence of thalassemia ranged from 8.16% to 34.35% in Hainan, Wuzhishan (34.35%), Baoting (31.46%), Qiongzhong (29.73%), Baisha (28.16%), Ledong (22.09%), Lingshui (21.02%), and Sanya (20.59%) have a higher prevalence than other areas. The results are shown in Figure 1.

## Discussion

### Molecular Epidemiological Characteristics of Thalassemia Genotypes in Childbearing Age Population of Hainan

In this study, we screened 231,596 individuals of the childbearing age population from the whole Hainan province. Totally, 31,780 (13.72%) subjects were diagnosed as carriers of thalassemia, and the frequencies of α-thalassemia, β-thalassemia, and α+β-thalassemia were 11.04%, 1.48%, and 1.20%, respectively. The aforementioned data indicate that the prevalence of thalassemia among the childbearing age population in Hainan ranks second in China, only lower than that in Guangxi, which is consistent with our previous research conclusions. (Wang et al., 2022). We further analyzed the frequency of specific mutation in all α (or β) mutant chromosomes in Hainan. The results showed that the five most common mutations of α-thalassemia were -α^3.7^, -α^4.2^, --^SEA^, α^WS^α, and α^QS^α, which account for 99.15% of all α-thalassemia carriers. These results suggested that the molecular profile of α-thalassemia in Hainan was different from that which is previously depicted in southern China, such as Guangxi, Guangdong, and Fujian, where--^SEA^ and α^CS^α always had higher allele frequencies, and--^SEA^ usually has the highest allele frequency (He et al., 2018; Peng et al., 2021; Zhuang et al., 2021).

A total of 16 β-thalassemia mutations in both β- and α+β-thalassemia carriers were identified in the present study, and β^CD41/42 (-TTCT)^ was the most frequent β-thalassemia mutation with the allele frequency of 73.08%, which was similar to the former studies reported in other regions of China but with a higher frequency in Hainan. (He et al., 2018; Peng et al., 2021; Lai et al., 2017). Other common mutations of β-gene were β^-28(A>G)^ (10.84%), β^IVS-Ⅱ-654(C>T)^ (5.25%), β^CD71/72 (+A)^ (3.96%), β^CD17(A>T)^ (3.62%), and β^CD26 (GAG>AAG)^ (1.71%). The ranking of the high-frequent genotypes was also different from other regions of China (Peng et al., 2021; He et al., 2017; Zhuang et al., 2021). In addition, this study also identified some uncommon thalassemia genotypes in the Chinese population. The fusion gene has resulted from a fusion between the α2 and ψα1 genes, and the genotype of Fusion/--^SEA^ can cause HbH disease (Huang et al., 2013). A total of 32 fusion gene carriers were detected; when the fusion gene was combined with the α^+^-gene, the hemoglobin level of the carriers was normal or slightly decreased, presenting an α-thalassemia minor phenotype. Both HKαα and αααanti4.2 were resulting from the unequal rearrangement in the homologous sequences of α-gene clusters and maintained intact function of the α-genes. So the heterozygote had no α-thalassemia phenotype (Shang et al., 2013). --^THAI^ is caused by the deletion of a large fragment of the α-gene, and the hematologic phenotype of carriers presents microcytosis (Eng et al., 2000). α^IVS-II-55 (T->G)^α and α^IVS-II-119 (-G, +CTCGGCCC)^α were rare mutations in the α2-globin gene and presumably neutral polymorphisms (https://www.ithanet.eu/db/ithagenes). An adult pregnant woman with -α^3.7^/α^IVS-II-55 (T->G), IVS-II-119 (-G, +CTCGGCCC)^α was detected in our study; the hematological phenotypic analysis showed that the Hb concentration was 102 g/L, MCV 71.7 fL, and MCH 21.6 pg. One adult male with -α^4.2^/α^IVS-II-55 (T->G),IVS-II-119 (-G, +CTCGGCCC)^α was also found in our study, and the subject had a normal hematologic phenotype. The mutation of β^-50 (G>A)^ located on the promoter of the β-gene and heterozygotes of carriers had normal hematological parameters (Zhao et al., 2020). In our study, one subject with the genotype of α^WS^α/-α^4.2^ combined β^-50(G>A)^/β^N^ and another with -α^3.7^/-α^4.2^ combined β^-50(G>A)^/β^N^ were detected, and the hematology indicators of both carriers showed the α-thalassemia minor phenotype. The mutation of β^IVS-Ⅱ-81 (C>T)^ is located in introns of the β-genes, and it is presumably a neutral polymorphism (https://www.ithanet.eu/db/ithagenes). One subject with the genotype of β^IVS-I-1 (G>T)^/β^IVS-Ⅱ-81 (C>T)^ and another with β^5’UTR;+40–43 (A>C)^/β^IVS-Ⅱ-81 (C>T)^ were also identified. The hematological indicators of β^IVS-I-1 (G>T)^/β^IVS-Ⅱ-81 (C>T)^ showed mild small-cell hypopigmented anemia, while the subject with β^5’UTR;+40–43 (A>C)^/β^IVS-Ⅱ-81 (C>T)^ had normal hematological parameters. This information may be valuable for genetic counseling of couples in high-prevalence areas of thalassemia.

### Molecular Epidemiological Characteristics of Thalassemia Genotypes in Han and Li Nationalities of Childbearing Age Population in Hainan

A total of 31,780 thalassemia carriers were detected in this study, among which 59.13% were Han people, 38.89% were Li people, and 1.98% were from other small ethnic groups. Due to the small number of subjects included in the total screening of Miao, Zhuang, and Hui, the prevalence of thalassemia in those groups may be inaccurate. Therefore, we mainly compared the molecular epidemiological characteristics of thalassemia in Han and Li populations. The results showed that the prevalence of thalassemia in the Li population (47.03%) was much higher than that in Han (9.37%). Except for β-thalassemia (1.31% of Li vs. 1.47% of Han), the prevalences of α-thalassemia (39.59% of Li vs.7.35% of Han) and α+β-thalassemia (6.13% of Li vs.0.56% of Han) in the Li population were significantly higher than those in Han, and the ranking of the high-frequent genotypes of α-thalassemia was different between the two nationalities. The frequency of thalassemia in the Li population obtained in our study was lower than that in the previous small-scale studies due to the enlarged sample size or different selection criteria for study subjects (Yao et al., 2014). These results indicated that the Li nationality living in Hainan has the highest prevalence of thalassemia genes in China. The reason may be that malaria occurs most frequently in the tropics, while the high prevalence of thalassemia is the result of natural selection by malaria. Therefore, on the only tropical island of China, the prevalence of thalassemia in the Li nationality, the earliest inhabitant of Hainan Island, was significantly higher than that of the Han nationality in Hainan and other parts of China. However, when the thalassemia prevalence of the Han people on Hainan was compared to that of the Han people in other parts of southern China, such as Guangdong and Guangxi, the frequency of thalassemia did not significantly increase. By investigating the timeline of the change of the population composition in the Hainan Island, we found that the Han population on the Hainan Island was only 2,295,691 in 1953 (http://www.hnszw.org.cn/data/news/2011/04/48961/) but increased to 8,498,241 in 2020 (http://stats.hainan.gov.cn/tjj/ztzl/rkpc/pcyw/202105/t20210512_2977751.html); the population of the Han increased nearly fourfold. With the development of Hainan, we believe that a large proportion of Han population in Hainan Island had migrated from other provinces of southern China, so the prevalence of thalassemia in the Han population in Hainan is similar to that of the Han people in other high-incidence areas in China. The ranking of the high-frequent genotypes of α-thalassemia was different between the two nationalities. The high-frequent genotypes of α-thalassemia in the Han were αα/--^SEA^ (25.55%), -α^3.7^/αα (22.17%), -α^4.2^/αα (21.59%), α^WS^α/αα (8.93%), -α^3.7^/-α^4.2^ (4.17%), and α^QS^α/αα (3.96%), and those of Li were -α^4.2^/αα (17.24%), -α^3.7^/αα (17.16%), -α^3.7^/-α^4.2^ (15.09%), α^WS^α/αα (9.69%), α^WS^α/-α^3.7^ (8.06%), -α^3.7^/-α^3.7^ (7.45%), αWSα/-α4.2 (7.13%), and -α^4.2^/-α^4.2^ (7.11%). The αα/--^SEA^ was the highest genotype of α-thalassemia in the Han nationality, which was similar to the reports from other provinces in China (He et al., 2018; Peng et al., 2021; Zhuang et al., 2021). However, αα/--^SEA^ only accounts for 1.87% of α-thalassemia in the Li nationality, far lower than other high-frequent genotypes, which was the biggest difference between the Li nationality and the Han nationality. For β-thalassemia, the ranking of high-frequent genotypes in both Han and Li was basically the same, but the frequency of β^CD41/42(-TTCT)^/β^N^ of the Li nationality was 90.96%, which was much higher than that of the Han nationality (56.32%) and the data reported in other provinces of southern China.

By analyzing the frequency of specific mutation in all α (or β) mutant chromosomes in both Han and Li nationalities, we found that the ranking of the high-frequent mutations of α-gene was significantly different between the two nationalities. Among the six common mutations of α-thalassemia in Chinese population, the high-frequent gene mutations of Han were -α^3.7^ (29.47%), -α^4.2^ (28.76%), --^SEA^ (23.52%), α^WS^α (12.95%), α^QS^α (3.97%), and α^CS^α (1.13%), and those of Li were -α^3.7^ (36.67%), -α^4.2^ (35.69%), α^WS^α (21.32%), α^QS^α (3.53%), --^SEA^ (2.50%), and α^CS^α (0.18%) in turn. The prominent difference between the two nationalities was that the allele frequency of--^SEA^ in the Li population was significantly lower than that in Han. For β-thalassemia and α + β-thalassemia, the mutation of CD41/42(−TTCT) had the highest allele frequency in both Han (62.17%) and Li (96.73%). The aforementioned results suggested that when Li people marry Li people, the risk of having children with severe α-thalassemia is lower than that of Han, while the risk of having children with severe β-thalassemia is higher than that of Han. Moreover, the regional differences in the prevalence of thalassemia in Hainan Island were mainly caused by the distribution of different ethnic populations. The prevalence of thalassemia in the settlement of the Li ethnic group, such as Wuzhishan, Baoting, Qiongzhong, and Baisha, was higher than that of other areas in the Hainan Island. Therefore, special attention should be paid to regional and ethnic differences in the prevention and control of thalassemia in Hainan.

## Conclusion

In this study, we revealed the characteristics of ethnic and regional differences in the prevalence of thalassemia in the childbearing age population of Hainan for the first time. The findings suggested that the Li nationality living in Hainan has the highest prevalence of thalassemia genes in China. Those findings will be useful for genetic counseling and the prevention of thalassemia.

## Data Availability

The original contributions presented in the study are included in the article/Supplementary Material; further inquiries can be directed to the corresponding authors.

## Abbreviations

CD, codon; MCV, mean corpuscular volume; Hb CS, Hb constant spring; Hb QS, Hb Quong Sze; Hb WS, Hb Westmead; SEA, Southeast Asian; EDTA, ethylenediaminetetraacetic acid; βN, βNormal; DNA, deoxyribonucleic acid; IVS, intervening sequences; UTR, untranslated region; Init, initiation.
