# Supplementary material for: Prevalence and Genetic Analysis of Thalassemia and Hemoglobinopathy in Different Ethnic Groups and Regions in Hainan Island, Southeast China
Source: Front Genet. 2022 Jun 13;13:874624. doi: 10.3389/fgene.2022.874624 (PMC9245582; doi:10.3389/fgene.2022.874624)
Supplement: Supplementary file 1 [file DataSheet1.docx]

**Table S1.** Molecular mutation spectrum of α+β-thalassemia in population of childbearing age in Hainan Island.

| **α+β-thalassemia genotype** | | | **Number of cases (n)** | **Constituent ratio (%)** |
| --- | --- | --- | --- | --- |
| **αα/--^SEA^** | **+** | **β^CD17^/β^N^** | **12** | **0.43** |
| **-α^3.7^/αα** | **+** | **β^CD17^/β^N^** | **11** | **0.39** |
| **-α^4.2^/--^SEA^** | **+** | **β^CD17^/β^N^** | **1** | **0.04** |
| **-α^4.2^/αα** | **+** | **β^CD17^/β^N^** | **6** | **0.22** |
| **α^WS^α/αα** | **+** | **β^CD17^/β^N^** | **6** | **0.22** |
| **α^CS^α/αα** | **+** | **β^CD17^/β^N^** | **3** | **0.11** |
| **-α^3.7^/αα** | **+** | **β^CD27/28^/β^N^** | **1** | **0.04** |
| **α^WS^α/αα** | **+** | **β^CD27/28^/β^N^** | **1** | **0.04** |
| **αα/--^SEA^** | **+** | **β^-28^/β^N^** | **20** | **0.72** |
| **-α^3.7^/--^SEA^** | **+** | **β^-28^/β^N^** | **2** | **0.07** |
| **-α^4.2^/-α^4.2^** | **+** | **β^-28^/β^N^** | **1** | **0.04** |
| **-α^3.7^/αα** | **+** | **β^-28^/β^N^** | **31** | **1.11** |
| **-α^4.2^/--^SEA^** | **+** | **β^-28^/β^N^** | **1** | **0.04** |
| **-α^4.2^/αα** | **+** | **β^-28^/β^N^** | **28** | **1.01** |
| **α^CS^α/αα** | **+** | **β^-28^/β^N^** | **2** | **0.07** |
| **α^WS^α/-α^4.2^** | **+** | **β^-28^/β^N^** | **1** | **0.04** |
| **α^WS^α/αα** | **+** | **β^-28^/β^N^** | **20** | **0.72** |
| **α^WS^α/--^SEA^** | **+** | **β^-28^/β^N^** | **2** | **0.07** |
| **α^QS^α/αα** | **+** | **β^-28^/β^N^** | **5** | **0.18** |
| **HKαα/αα** | **+** | **β^-28^/β^N^** | **2** | **0.07** |
| **-α^3.7^/αα** | **+** | **β^-29^/β^N^** | **3** | **0.11** |
| **-α^4.2^/αα** | **+** | **β^-29^/β^N^** | **1** | **0.04** |
| **αα/--^SEA^** | **+** | **β^CD41-42^/β^N^** | **113** | **4.06** |
| **-α^3.7^/--^SEA^** | **+** | **β^CD41-42^/β^N^** | **12** | **0.43** |
| **-α^3.7^/-α^3.7^** | **+** | **β^CD41-42^/β^N^** | **121** | **4.34** |
| **-α^3.7^/-α^4.2^** | **+** | **β^CD41-42^/β^N^** | **231** | **8.29** |
| **-α^3.7^/αα** | **+** | **β^CD41-42^/β^N^** | **557** | **20.00** |
| **-α^4.2^/--^SEA^** | **+** | **β^CD41-42^/β^N^** | **7** | **0.25** |
| **-α^4.2^/-α^4.2^** | **+** | **β^CD41-42^/β^N^** | **116** | **4.17** |
| **-α^4.2^/αα** | **+** | **β^CD41-42^/β^N^** | **534** | **19.17** |
| **α^CS^α/-α^3.7^** | **+** | **β^CD41-42^/β^N^** | **2** | **0.07** |
| **α^CS^α/-α^4.2^** | **+** | **β^CD41-42^/β^N^** | **1** | **0.04** |
| **α^CS^α/αα** | **+** | **β^CD41-42^/β^N^** | **7** | **0.25** |
| **α^QS^α/-α^3.7^** | **+** | **β^CD41-42^/β^N^** | **21** | **0.75** |
| **α^QS^α/-α^4.2^** | **+** | **β^CD41-42^/β^N^** | **21** | **0.75** |
| **α^QS^α/αα** | **+** | **β^CD41-42^/β^N^** | **33** | **1.18** |
| **α^WS^α/--^SEA^** | **+** | **β^CD41-42^/β^N^** | **15** | **0.54** |
| **α^WS^α/-α^3.7^** | **+** | **β^CD41-42^/β^N^** | **137** | **4.92** |
| **α^WS^α/-α^4.2^** | **+** | **β^CD41-42^/β^N^** | **133** | **4.78** |
| **α^QS^α/α^WS^α** | **+** | **β^CD41-42^/β^N^** | **11** | **0.39** |
| **α^WS^α/αα** | **+** | **β^CD41-42^/β^N^** | **361** | **12.96** |
| **α^WS^α/α^WS^α** | **+** | **β^CD41-42^/β^N^** | **50** | **1.80** |
| **α^QS^α/--^SEA^** | **+** | **β^CD41-42^/β^N^** | **1** | **0.04** |
| **Fusion/-α^4.2^** | **+** | **β^CD41-42^/β^N^** | **2** | **0.07** |
| **αααanti^4.2^/-α^3.7^** | **+** | **β^CD41-42^/β^N^** | **1** | **0.04** |
| **-α^3.7^/αα** | **+** | **β^CD43^/β^N^** | **4** | **0.14** |
| **-α^4.2^/αα** | **+** | **β^CD43^/β^N^** | **1** | **0.04** |
| **α^QS^α/αα** | **+** | **β^CD43/^β^N^** | **1** | **0.04** |
| **αα/--^SEA^** | **+** | **β^IVS-Ⅱ-654^/β^N^** | **17** | **0.61** |
| **-α^3.7^/--^SEA^** | **+** | **β^IVS-Ⅱ-654^/β^N^** | **1** | **0.04** |
| **-α^3.7^/αα** | **+** | **β^IVS-Ⅱ-654^/β^N^** | **9** | **0.32** |
| **-α^4.2^/αα** | **+** | **β^IVS-Ⅱ-654^/β^N^** | **10** | **0.36** |
| **α^CS^α/αα** | **+** | **β^IVS-Ⅱ-654^/β^N^** | **1** | **0.04** |
| **α^QS^α/αα** | **+** | **β^IVS-Ⅱ-654^/β^N^** | **3** | **0.11** |
| **α^WS^α/αα** | **+** | **β^IVS-Ⅱ-654^/β^N^** | **6** | **0.22** |
| **α^WS^α/-α^4.2^** | **+** | **β^IVS-Ⅱ-654^/β^N^** | **2** | **0.07** |
| **αα/--^SEA^** | **+** | **β^CD71-72^/β^N^** | **7** | **0.25** |
| **-α^3.7^/-α^3.7^** | **+** | **β^CD71-72^/β^N^** | **2** | **0.07** |
| **-α^3.7^/-α^4.2^** | **+** | **β^CD71-72^/β^N^** | **1** | **0.04** |
| **-α^3.7^/αα** | **+** | **β^CD71-72^/β^N^** | **15** | **0.54** |
| **-α^4.2^/-α^4.2^** | **+** | **β^CD71-72^/β^N^** | **1** | **0.04** |
| **-α^4.2^/αα** | **+** | **β^CD71-72^/β^N^** | **19** | **0.68** |
| **α^WS^α/-α^3.7^** | **+** | **β^CD71-72^/β^N^** | **5** | **0.18** |
| **α^WS^α/αα** | **+** | **β^CD71-72^/β^N^** | **4** | **0.14** |
| **-α^3.7^/αα** | **+** | **β^5’UTR;+40-43^/β^N^** | **1** | **0.04** |
| **-α^4.2^/αα** | **+** | **β^5’UTR;+40-43^/β^N^** | **1** | **0.04** |
| **-α^3.7^/--^SEA^** | **+** | **β^5’UTR;+40-43^/β^N^** | **1** | **0.04** |
| **αα/--^SEA^** | **+** | **β^Init CD^/β^N^** | **1** | **0.04** |
| **-α^3.7^/αα** | **+** | **β^Init CD^/β^N^** | **1** | **0.04** |
| **-α^4.2^/αα** | **+** | **β^Init CD^/β^N^** | **1** | **0.04** |
| **α^WS^α/αα** | **+** | **β^Init CD^/β^N^** | **1** | **0.04** |
| **-α^3.7^/αα** | **+** | **β^IVS-I-1 (G＞T)^/β^N^** | **1** | **0.04** |
| **αα/--^SEA^** | **+** | **β^CD26^/β^N^** | **2** | **0.07** |
| **-α^3.7^/αα** | **+** | **β^CD26^/β^N^** | **6** | **0.22** |
| **-α^4.2^/αα** | **+** | **β^CD26^/β^N^** | **4** | **0.14** |
| **α^WS^α/αα** | **+** | **β^CD26^/β^N^** | **2** | **0.07** |
| **α^WS^α/α^WS^α** | **+** | **β^CD26^/β^N^** | **1** | **0.04** |
| **α^WS^α/-α^4.2^** | **+** | **β^-50^/β^N^** | **1** | **0.04** |
| **-α^3.7^/-α^4.2^** | **+** | **β^-50^/β^N^** | **1** | **0.04** |
| **α^CS^α/αα** | **+** | **β^CD41-42^/β^-28^** | **1** | **0.04** |
| **-α^4.2^/αα** | **+** | **β^CD41-42^/β^-28^** | **1** | **0.04** |
| **αα/--^SEA^** | **+** | **β^CD41-42^/β^-28^** | **1** | **0.04** |
| **αα/--^SEA^** | **+** | **β^-28^/β^IVS-Ⅱ-654^** | **1** | **0.04** |
| **Total** | | | **2785** | **100.00** |

**Table S2.** Molecular mutation spectrum of α+β-thalassemia in Han population of childbearing age in Hainan Island.

| **α+β thalassemia genotype** | | | **Number of cases (n)** | **Constituent ratio (%)** |
| --- | --- | --- | --- | --- |
| **αα/--^SEA^** | **+** | **β^CD17^/β^N^** | **11** | **0.98** |
| **-α^3.7^/αα** | **+** | **β^CD17^/β^N^** | **9** | **0.81** |
| **-α^4.2^/αα** | **+** | **β^CD17^/β^N^** | **4** | **0.36** |
| **α^WS^α/αα** | **+** | **β^CD17^/β^N^** | **4** | **0.36** |
| **α^CS^α/αα** | **+** | **β^CD17^/β^N^** | **2** | **0.18** |
| **-α^3.7^/αα** | **+** | **β^CD27/28^/β^N^** | **1** | **0.09** |
| **α^WS^α/αα** | **+** | **β^CD27/28^/β^N^** | **1** | **0.09** |
| **αα/--^SEA^** | **+** | **β^-28^/β^N^** | **19** | **1.70** |
| **-α^3.7^/--^SEA^** | **+** | **β^-28^/β^N^** | **2** | **0.18** |
| **-α^4.2^/-α^4.2^** | **+** | **β^-28^/β^N^** | **1** | **0.09** |
| **-α^3.7^/αα** | **+** | **β^-28^/β^N^** | **28** | **2.51** |
| **-α^4.2^/--^SEA^** | **+** | **β^-28^/β^N^** | **1** | **0.09** |
| **-α^4.2^/αα** | **+** | **β^-28^/β^N^** | **27** | **2.42** |
| **α^CS^α/αα** | **+** | **β^-28^/β^N^** | **2** | **0.18** |
| **α^WS^α/αα** | **+** | **β^-28^/β^N^** | **15** | **1.34** |
| **α^WS^α/--^SEA^** | **+** | **β^-28^/β^N^** | **1** | **0.09** |
| **α^QS^α/αα** | **+** | **β^-28^/β^N^** | **5** | **0.45** |
| **HKαα/αα** | **+** | **β^-28^/β^N^** | **2** | **0.18** |
| **-α^3.7^/αα** | **+** | **β^-29^/β^N^** | **3** | **0.27** |
| **-α^4.2^/αα** | **+** | **β^-29^/β^N^** | **1** | **0.09** |
| **αα/--^SEA^** | **+** | **β^CD41-42^/β^N^** | **87** | **7.79** |
| **-α^3.7^/--^SEA^** | **+** | **β^CD41-42^/β^N^** | **2** | **0.18** |
| **-α^3.7^/-α^3.7^** | **+** | **β^CD41-42^/β^N^** | **20** | **1.79** |
| **-α^3.7^/-α^4.2^** | **+** | **β^CD41-42^/β^N^** | **43** | **3.85** |
| **-α^3.7^/αα** | **+** | **β^CD41-42^/β^N^** | **233** | **20.86** |
| **-α^4.2^/--^SEA^** | **+** | **β^CD41-42^/β^N^** | **4** | **0.36** |
| **-α^4.2^/-α^4.2^** | **+** | **β^CD41-42^/β^N^** | **28** | **2.51** |
| **-α^4.2^/αα** | **+** | **β^CD41-42^/β^N^** | **212** | **18.98** |
| **α^CS^α/-α^3.7^** | **+** | **β^CD41-42^/β^N^** | **2** | **0.18** |
| **α^CS^α/-α^4.2^** | **+** | **β^CD41-42^/β^N^** | **1** | **0.09** |
| **α^CS^α/αα** | **+** | **β^CD41-42^/β^N^** | **5** | **0.45** |
| **α^QS^α/-α^3.7^** | **+** | **β^CD41-42^/β^N^** | **3** | **0.27** |
| **α^QS^α/-α^4.2^** | **+** | **β^CD41-42^/β^N^** | **1** | **0.09** |
| **α^QS^α/αα** | **+** | **β^CD41-42^/β^N^** | **13** | **1.16** |
| **α^WS^α/--^SEA^** | **+** | **β^CD41-42^/β^N^** | **2** | **0.18** |
| **α^WS^α/-α^3.7^** | **+** | **β^CD41-42^/β^N^** | **27** | **2.42** |
| **α^WS^α/-α^4.2^** | **+** | **β^CD41-42^/β^N^** | **28** | **2.51** |
| **α^QS^α/α^WS^α** | **+** | **β^CD41-42^/β^N^** | **2** | **0.18** |
| **α^WS^α/αα** | **+** | **β^CD41-42^/β^N^** | **137** | **12.26** |
| **α^WS^α/α^WS^α** | **+** | **β^CD41-42^/β^N^** | **13** | **1.16** |
| **α^QS^α/--^SEA^** | **+** | **β^CD41-42^/β^N^** | **1** | **0.09** |
| **Fusion/-α^4.2^** | **+** | **β^CD41-42^/β^N^** | **1** | **0.09** |
| **αααanti^4.2^/-α^3.7^** | **+** | **β^CD41-42^/β^N^** | **1** | **0.09** |
| **-α^3.7^/αα** | **+** | **β^CD43^/β^N^** | **4** | **0.36** |
| **-α^4.2^/αα** | **+** | **β^CD43^/β^N^** | **1** | **0.09** |
| **α^QS^α/αα** | **+** | **β^CD43/^β^N^** | **1** | **0.09** |
| **αα/--^SEA^** | **+** | **β^IVS-Ⅱ-654^/β^N^** | **16** | **1.43** |
| **-α^3.7^/--^SEA^** | **+** | **β^IVS-Ⅱ-654^/β^N^** | **1** | **0.09** |
| **-α^3.7^/αα** | **+** | **β^IVS-Ⅱ-654^/β^N^** | **7** | **0.63** |
| **-α^4.2^/αα** | **+** | **β^IVS-Ⅱ-654^/β^N^** | **10** | **0.90** |
| **α^QS^α/αα** | **+** | **β^IVS-Ⅱ-654^/β^N^** | **3** | **0.27** |
| **α^WS^α/αα** | **+** | **β^IVS-Ⅱ-654^/β^N^** | **3** | **0.27** |
| **α^WS^α/-α^4.2^** | **+** | **β^IVS-Ⅱ-654^/β^N^** | **1** | **0.09** |
| **αα/--^SEA^** | **+** | **β^CD71-72^/β^N^** | **5** | **0.45** |
| **-α^3.7^/αα** | **+** | **β^CD71-72^/β^N^** | **14** | **1.25** |
| **-α^4.2^/αα** | **+** | **β^CD71-72^/β^N^** | **17** | **1.52** |
| **α^WS^α/-α^3.7^** | **+** | **β^CD71-72^/β^N^** | **2** | **0.18** |
| **α^WS^α/αα** | **+** | **β^CD71-72^/β^N^** | **3** | **0.27** |
| **-α^3.7^/αα** | **+** | **β^5’UTR;+40-43^/β^N^** | **1** | **0.09** |
| **-α^3.7^/--^SEA^** | **+** | **β^5’UTR;+40-43^/β^N^** | **1** | **0.09** |
| **-α^4.2^/αα** | **+** | **β^5’UTR;+40-43^/β^N^** | **1** | **0.09** |
| **αα/--^SEA^** | **+** | **β^Init CD^/β^N^** | **1** | **0.09** |
| **-α^3.7^/αα** | **+** | **β^Init CD^/β^N^** | **1** | **0.09** |
| **-α^4.2^/αα** | **+** | **β^Init CD^/β^N^** | **1** | **0.09** |
| **α^WS^α/αα** | **+** | **β^Init CD^/β^N^** | **1** | **0.09** |
| **-α^3.7^/αα** | **+** | **β^IVS-I-1 (G＞T)^/β^N^** | **1** | **0.09** |
| **-α^3.7^/αα** | **+** | **β^CD26^/β^N^** | **5** | **0.45** |
| **-α^4.2^/αα** | **+** | **β^CD26^/β^N^** | **4** | **0.36** |
| **αα/--^SEA^** |  | **β^CD26^/β^N^** | **2** | **0.18** |
| **α^WS^α/α^WS^α** | **+** | **β^CD26^/β^N^** | **1** | **0.09** |
| **-α^4.2^/αα** | **+** | **β^CD41-42^/β^-28^** | **1** | **0.09** |
| **α^CS^α/αα** | **+** | **β^CD41-42^/β^-28^** | **1** | **0.09** |
| **αα/--^SEA^** | **+** | **β^CD41-42^/β^-28^** | **1** | **0.09** |
| **αα/--^SEA^** | **+** | **β^-28^/β^IVS-Ⅱ-654^** | **1** | **0.09** |
| **Total** | | | **1117** | **100.00** |

**Table S3.** Molecular mutation spectrum of α+β-thalassemia in Li population of childbearing age in Hainan Island.

| **α+β thalassemia genotype** | | | **Number of cases (n)** | **Constituent ratio (%)** |
| --- | --- | --- | --- | --- |
| **-α^3.7^/αα** | **+** | **β^-28^/β^N^** | **3** | **0.19** |
| **-α^4.2^/αα** | **+** | **β^-28^/β^N^** | **1** | **0.06** |
| **α^WS^α/-α^4.2^** | **+** | **β^-28^/β^N^** | **1** | **0.06** |
| **α^WS^α/αα** | **+** | **β^-28^/β^N^** | **5** | **0.31** |
| **α^WS^α/--^SEA^** | **+** | **β^-28^/β^N^** | **1** | **0.06** |
| **αα/--^SEA^** | **+** | **β^CD41-42^/β^N^** | **12** | **0.74** |
| **-α^3.7^/--^SEA^** | **+** | **β^CD41-42^/β^N^** | **9** | **0.56** |
| **-α^3.7^/-α^3.7^** | **+** | **β^CD41-42^/β^N^** | **101** | **6.27** |
| **-α^3.7^/-α^4.2^** | **+** | **β^CD41-42^/β^N^** | **188** | **11.66** |
| **-α^3.7^/αα** | **+** | **β^CD41-42^/β^N^** | **312** | **19.35** |
| **-α^4.2^/--^SEA^** | **+** | **β^CD41-42^/β^N^** | **3** | **0.19** |
| **-α^4.2^/-α^4.2^** | **+** | **β^CD41-42^/β^N^** | **87** | **5.40** |
| **-α^4.2^/αα** | **+** | **β^CD41-42^/β^N^** | **317** | **19.67** |
| **α^QS^α/-α^3.7^** | **+** | **β^CD41-42^/β^N^** | **18** | **1.12** |
| **α^QS^α/-α^4.2^** | **+** | **β^CD41-42^/β^N^** | **20** | **1.24** |
| **α^QS^α/αα** | **+** | **β^CD41-42^/β^N^** | **20** | **1.24** |
| **α^WS^α/--^SEA^** | **+** | **β^CD41-42^/β^N^** | **13** | **0.81** |
| **α^WS^α/-α^3.7^** | **+** | **β^CD41-42^/β^N^** | **110** | **6.82** |
| **α^WS^α/-α^4.2^** | **+** | **β^CD41-42^/β^N^** | **105** | **6.51** |
| **α^QS^α/α^WS^α** | **+** | **β^CD41-42^/β^N^** | **9** | **0.56** |
| **α^WS^α/αα** | **+** | **β^CD41-42^/β^N^** | **217** | **13.46** |
| **α^WS^α/α^WS^α** | **+** | **β^CD41-42^/β^N^** | **37** | **2.30** |
| **Fusion/-α^4.2^** | **+** | **β^CD41-42^/β^N^** | **1** | **0.06** |
| **αα/--^SEA^** | **+** | **β^IVS-Ⅱ-654^/β^N^** | **1** | **0.06** |
| **-α^3.7^/αα** | **+** | **β^IVS-Ⅱ-654^/β^N^** | **2** | **0.12** |
| **α^WS^α/αα** | **+** | **β^IVS-Ⅱ-654^/β^N^** | **3** | **0.19** |
| **α^WS^α/-α^4.2^** | **+** | **β^IVS-Ⅱ-654^/β^N^** | **1** | **0.06** |
| **-α^3.7^/-α^3.7^** | **+** | **β^CD71-72^/β^N^** | **2** | **0.12** |
| **-α^4.2^/-α^4.2^** | **+** | **β^CD71-72^/β^N^** | **1** | **0.06** |
| **-α^3.7^/αα** | **+** | **β^CD71-72^/β^N^** | **1** | **0.06** |
| **-α^4.2^/αα** | **+** | **β^CD71-72^/β^N^** | **2** | **0.12** |
| **-α^3.7^/-α^4.2^** | **+** | **β^CD71-72^/β^N^** | **1** | **0.06** |
| **α^WS^α/-α^3.7^** | **+** | **β^CD71-72^/β^N^** | **3** | **0.19** |
| **α^WS^α/αα** | **+** | **β^CD71-72^/β^N^** | **1** | **0.06** |
| **α^WS^α/αα** | **+** | **β^βE^/β^N^** | **2** | **0.12** |
| **α^WS^α/-α^4.2^** | **+** | **β^-50^/β^N^** | **1** | **0.06** |
| **-α^3.7^/-α^4.2^** | **+** | **β^-50^/β^N^** | **1** | **0.06** |
| **Total** | | | **1612** | **100.00** |

**Table S4.** Molecular mutation spectrum of α-thalassemia in Miao, Zhuang and Hui population of childbearing age in Hainan Island.

| **α-thalassemia genotype** | **T**ype | **Cases of Miao (n)** | **Frequency of Miao (%)** | **Cases of**  **Zhuang (n)** | **Frequency of**  **Zhuang (%)** | **Cases of**  **Hui (n)** | **Frequency of**  **Hui (%)** |
| --- | --- | --- | --- | --- | --- | --- | --- |
|  |  |  |  |  |  |  |  |
| **α**α/--SEA | **α**0/α | **91** | **39.22** | **43** | **47.25** | **3** | **7.69** |
| **-α**3.7/--SEA | **α**+/α0 | **6** | **2.59** | **0** | **0.00** | **1** | **2.56** |
| **-α**3.7/-α3.7 | **α**+/α+ | **5** | **2.16** | **0** | **0.00** | **1** | **2.56** |
| **-α**3.7/-α4.2 | **α**+/α+ | **2** | **0.86** | **1** | **1.10** | **0** | **0.00** |
| **-α**3.7/αα | **α**+/α | **51** | **21.98** | **14** | **15.38** | **16** | **41.03** |
| **-α**4.2/--SEA | **α**+/α0 | **3** | **1.29** | **1** | **1.10** | **0** | **0.00** |
| **-α**4.2/-α4.2 | **α**+/α+ | **4** | **1.72** | **0** | **0.00** | **0** | **0.00** |
| **-α**4.2/αα | **α**+/α | **25** | **10.78** | **10** | **10.99** | **4** | **10.26** |
| **α**CSα/--SEA | **α**+/α0 | **0** | **0.00** | **1** | **1.10** | **0** | **0.00** |
| **α**CSα/-α3.7 | **α**+/α+ | **1** | **0.43** | **0** | **0.00** | **0** | **0.00** |
| **α**CSα/αα | **α**+/α | **4** | **1.72** | **6** | **6.59** | **0** | **0.00** |
| **α**QSα/αα | **α**+/α | **3** | **1.29** | **1** | **1.10** | **0** | **0.00** |
| **α**WSα/--SEA | **α**+/α0 | **5** | **2.16** | **1** | **1.10** | **0** | **0.00** |
| **α**WSα/-α3.7 | **α**+/α+ | **1** | **0.43** | **0** | **0.00** | **0** | **0.00** |
| **α**WSα/-α4.2 | **α**+/α+ | **2** | **0.86** | **0** | **0.00** | **1** | **2.56** |
| **α**WSα/αα | **α**+/α | **27** | **11.64** | **13** | **14.29** | **13** | **33.33** |
| **α**CSα/αWSα | **α**+/α+ | **1** | **0.43** | **0** | **0.00** | **0** | **0.00** |
| **α**WSα/αWSα | **α**+/α+ | **1** | **0.43** | **0** | **0.00** | **0** | **0.00** |
| **Total** |  | **232** | **100.00** | **91** | **100.00** | **39** | **100.00** |

**Table S5.** Molecular mutation spectrum of β-thalassemia in Miao, Zhuang and Hui population of childbearing age in Hainan Island.

| **β-thalassemia genotype** | **Type** | **Cases of**  **Miao (n)** | **Frequency of**  **Miao (%)** | **Cases of**  **Zhuang (n)** | **Frequency of**  **Zhuang (%)** | **Cases of**  **Hui (n)** | **Frequency of**  **Hui (%)** |
| --- | --- | --- | --- | --- | --- | --- | --- |
|  |  |  |  |  |  |  |  |
| **β**CD17 (A＞T)**/β**N | **β**0**/β** | **14** | **21.88** | **8** | **22.22** | **0** | **0.00** |
| **β**CD27/28 (+C)**/β**N | **β**0**/β** | **0** | **0.00** | **0** | **0.00** | **1** | **12.50** |
| **β**-28 (A＞G)**/β**N | **β**+**/β** | **5** | **7.81** | **1** | **2.78** | **4** | **50.00** |
| **β**-29 (A＞G)**/β**N | **β**+**/β** | **0** | **0.00** | **2** | **5.56** | **0** | **0.00** |
| **β**CD41/42 (-TTCT)**/β**N | **β**0**/β** | **43** | **67.19** | **18** | **50.00** | **3** | **37.50** |
| **β**CD71/72 (+A)**/β**N | **β**0**/β** | **0** | **0.00** | **3** | **8.33** | **0** | **0.00** |
| **β**IVS-I-1 (G＞T)**/β**N | **β**0**/β** | **1** | **1.56** | **1** | **2.78** | **0** | **0.00** |
| **β**CD 26 (GAG＞AAG)**/β**N | **β**+**/β** | **0** | **0.00** | **3** | **8.33** | **0** | **0.00** |
| **β**CD27/28 (+C)**/β**& | **β**0**/β** | **1** | **1.56** | **0** | **0.00** | **0** | **0.00** |
| **Total** |  | **64** | **100.00** | **36** | **100.00** | **8** | **100.00** |

**Table S6.** Molecular mutation spectrum of α+β-thalassemia in Miao population of childbearing age in Hainan Island.

| **α+β-thalassemia genotype** | | | **Number of cases (n)** | **Constituent ratio (%)** |
| --- | --- | --- | --- | --- |
| **-α^3.7^/αα** | **+** | **β^CD17^/β^N^** | **1** | **2.94** |
| **-α^4.2^/αα** | **+** | **β^CD17^/β^N^** | **2** | **5.88** |
| **-α^4.2^/--^SEA^** | **+** | **β^CD17^/β^N^** | **1** | **2.94** |
| **αα/--^SEA^** | **+** | **β^CD41-42^/β^N^** | **9** | **26.47** |
| **-α^3.7^/--^SEA^** | **+** | **β^CD41-42^/β^N^** | **1** | **2.94** |
| **-α^3.7^/αα** | **+** | **β^CD41-42^/β^N^** | **10** | **29.41** |
| **-α^4.2^/-α^4.2^** | **+** | **β^CD41-42^/β^N^** | **1** | **2.94** |
| **-α^4.2^/αα** | **+** | **β^CD41-42^/β^N^** | **4** | **11.76** |
| **α^WS^α/αα** | **+** | **β^CD41-42^/β^N^** | **5** | **14.71** |
| **Total** | | | **34** | **100.00** |

**Table S7.** Molecular mutation spectrum of α+β-thalassemia in Zhuang population of childbearing age in Hainan Island.

| **α+β-thalassemia genotype** | | | **Number of cases (n)** | **Constituent ratio (%)** |
| --- | --- | --- | --- | --- |
| **α^WS^α/αα** | **+** | **β^CD17^/β^N^** | **1** | **14.29** |
| **αα/--^SEA^** | **+** | **β^-28^/β^N^** | **1** | **14.29** |
| **αα/--^SEA^** | **+** | **β^CD41-42^/β^N^** | **2** | **28.57** |
| **-α^4.2^/αα** | **+** | **β^CD41-42^/β^N^** | **1** | **14.29** |
| **αα/--^SEA^** | **+** | **β^CD71-72^/β^N^** | **2** | **28.57** |
| **Total** | | | **7** | **100.00** |

**Table S8.** Molecular mutation spectrum of α+β-thalassemia in Hui population of childbearing age in Hainan Island.

| **α+β-thalassemia genotype** | | | **Number of cases (n)** | **Constituent ratio (%)** |
| --- | --- | --- | --- | --- |
| **α^WS^α/αα** | **+** | **β^CD41-42^/β^N^** | **1** | **100.00** |
| **Total** | | | **1** | **100.00** |
